# Theta and Alpha Oscillations in Attentional Interaction during Distracted Driving

**DOI:** 10.3389/fnbeh.2018.00003

**Published:** 2018-02-09

**Authors:** Yu-Kai Wang, Tzyy-Ping Jung, Chin-Teng Lin

**Affiliations:** ^1^Centre for Artificial Intelligence, Faculty of Engineering and Information Technology, University of Technology Sydney, Sydney, NSW, Australia; ^2^Swartz Center for Computational Neuroscience, Institute for Neural Computation, University of California, San Diego, San Diego, CA, United States

**Keywords:** electroencephalogram (EEG), dual-task, stimulus onset asynchrony (SOA), interactive attention, independent component analysis (ICA), theta, alpha

## Abstract

Performing multiple tasks simultaneously usually affects the behavioral performance as compared with executing the single task. Moreover, processing multiple tasks simultaneously often involve more cognitive demands. Two visual tasks, lane-keeping task and mental calculation, were utilized to assess the brain dynamics through 32-channel electroencephalogram (EEG) recorded from 14 participants. A 400-ms stimulus onset asynchrony (SOA) factor was used to induce distinct levels of attentional requirements. In the dual-task conditions, the deteriorated behavior reflected the divided attention and the overlapping brain resources used. The frontal, parietal and occipital components were decomposed by independent component analysis (ICA) algorithm. The event- and response-related theta and alpha oscillations in selected brain regions were investigated first. The increased theta oscillation in frontal component and decreased alpha oscillations in parietal and occipital components reflect the cognitive demands and attentional requirements as executing the designed tasks. Furthermore, time-varying interactive over-additive (O-Add), additive (Add) and under-additive (U-Add) activations were explored and summarized through the comparison between the summation of the elicited spectral perturbations in two single-task conditions and the spectral perturbations in the dual task. Add and U-Add activations were observed while executing the dual tasks. U-Add theta and alpha activations dominated the posterior region in dual-task situations. Our results show that both deteriorated behaviors and interactive brain activations should be comprehensively considered for evaluating workload or attentional interaction precisely.

## Introduction

Humans are immersed in an overload of sensory information in their interaction with the environment. Meanwhile, cognitive functions allow us to integrate multiple streams of information from the complex world and may expedite estimation with more cognitive demands (Pashler, 1994). Performing multiple tasks leads to attentional interference in which humans must shift their attention between multiple operations (Mishra et al., 2013). Compared with the cost of attending to only one task, there might be extra behavioral costs when performing dual/multiple tasks simultaneously (Szameitat et al., 2011; Nijboer et al., 2014). Previous findings suggest that attentional interaction results in an increased reaction time (RT) or high error rate (Norman and Bobrow, 1975; Pashler, 1994; Lin et al., 2011), reflecting the overlapping resources used during dual situations or multitasking (Salvucci and Taatgen, 2008). Limited attention leads to a bottleneck when processing multiple cognitively demanding operations simultaneously (Pashler, 1994; Salvucci and Taatgen, 2008).

Due to impaired behavior and limited attention, the brain dynamics during the simultaneous processing of dual/multiple tasks have been assumed to show a rich activation. Szameitat et al. (2011) reported that activity during dual tasks was composed of not only the task-related activation but also the activation in a dual-task-specific region. This dual-task-specific activation suggests that there are mental efforts associated with managing tasks or willful attention during dual tasks (Frith and Dolan, 1996; Jaeggi et al., 2003). However, this dual-task-specific action has been debated (Moisala et al., 2015). Recent studies have shown that while handling dual or multiple tasks simultaneously, subjects did not exhibit activation of additional brain regions (Nijboer et al., 2014; Moisala et al., 2015). Instead of a specific or extra brain region associated with the dual-task situation, the regional dynamics during the dual tasks were composed of the activation induced by each single task (Adcock et al., 2000; Just et al., 2001; Jaeggi et al., 2003). Nijboer et al. (2014) summarized the dual-task dynamics into three categories, over-additive (O-Add), additive (Add) and under-additive (U-Add) activations, to characterize the interactive oscillations between the single- and dual-task conditions. The interactive activation in the dual task is higher than, equal to or lower than the summation of the activation associated with the specific single tasks in O-Add, Add or U-Add activation, respectively.

Most studies have explored the brain activation associated with interferential attention through the use of functional magnetic resonance imaging (fMRI) or positron emission tomography (PET; Adcock et al., 2000; Jaeggi et al., 2003; Spiers and Maguire, 2007; Just et al., 2008; Nijboer et al., 2014). During the performance of dual or multiple tasks, activation in the frontal and posterior regions has been reported in previous studies (Tachibana et al., 2012; Schweizer et al., 2013). However, monitoring and tracking attention-related brain dynamics is crucial in many applied fields, such as distracted driving in daily life. To measure attention, investigating activation during dual tasks through readily wearable devices is essential. Electroencephalogram (EEG) represents a neuroimaging technique used to noninvasively measure brain activity (Mullen et al., 2015; Wang et al., 2015; Huang et al., 2016). The stimulus onset asynchrony (SOA) factor denotes the amount of time between the two stimuli in the dual-task studies (Marois and Ivanoff, 2005; Lin et al., 2011). A short time interval between two stimuli may lead the distinct attentional requirements. EEG also has the high temporal resolution to investigate the distinct level of attentional interaction.

The participants performed experimental tasks included lane-keeping and mental calculation in the current study. Visual resources were involved in lane deviation (Jäncke et al., 2008; Calhoun and Pearlson, 2012; Lin et al., 2012a), and visual, memory and executive resources were predicted to be involved in solving the numerical equation (Fernández et al., 1995; Micheloyannis et al., 2005; Sammer et al., 2007; Lin et al., 2012b). Sustained attention and decision making have been mainly associated with frontal theta oscillations (Lin et al., 2011; Borghini et al., 2014). Alpha suppression in the posterior area, including the parietal and occipital regions, is also associated with attentional demands and cognitive progress (Klimesch et al., 1998; Foxe and Snyder, 2011; Lai et al., 2012). Through the experimental tasks and attentional demands, we focused on the brain dynamics in the frontal, parietal and occipital regions, which are highly correlated with attentional processes (Tachibana et al., 2012; Schweizer et al., 2013).

Our current study contributes three aspects to this field. First, we systematically explored the event-related and response-related perturbations in the frontal, parietal and occipital regions. In particular, we primarily focused on theta and alpha oscillations, which are involved in attentional demands (Klimesch, 1999). Second, we summarized the EEG dynamics during dual tasks as three specific types of activation under high temporal resolution. Third, we evaluated and investigated the time-varying interactive EEG theta and alpha activations during the performance of three dual tasks. In the present study, we hypothesized that the regional EEG dynamics during the performance of dual tasks would be composed of the activation induced by each single task. The stimulus- and response-related activation patterns induced by performing each single task were first thoroughly elucidated. These spectral perturbations were then investigated under the distinct dual-task conditions. Therefore, we compared the estimated and measured theta and alpha activation patterns to infer the attentional interaction during the dual conditions.

## Materials and Methods

### Participants

Fourteen college students (all males, mean age: 23.4, age range: 21–28), recruited from National Chiao-Tung University, Hsinchu, Taiwan, participated in the current study. This study was carried out in accordance with the recommendations of Taipei Veterans General Hospital with written informed consent from all subjects. The Institutional Review Board of the Taipei Veterans General Hospital approved the protocol. All right-handed participants had normal or corrected-to-normal vision and had driving experience of more than 1 year. The consumption of caffeine, tobacco, alcohol and drugs was prohibited before the experiment. They received a monetary reward for their participation upon finishing the entire experiment.

### Experimental Design and Procedure

Experiments for this study were conducted in a driving simulator. A real car was mounted on a dynamic 6-degrees-of-freedom motion platform, and 360° scenes were rendered by seven LCD projectors. In this immersive virtual reality scenario, the participants cruised at a fixed speed of 100 km/h on a highway throughout the experiment. Two tasks, lane-keeping and mental calculation, were utilized in this study.

**Figure 1:**
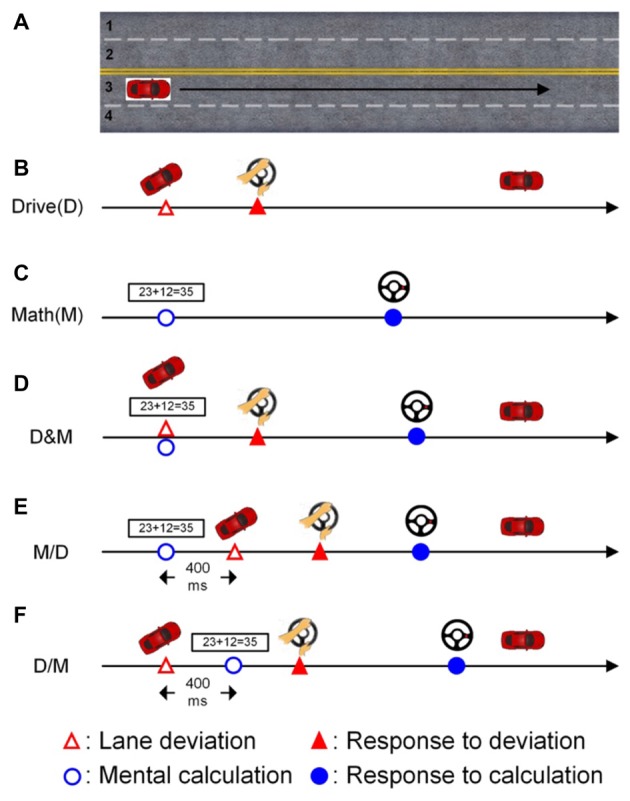
The scenarios of the single- and dual-task conditions. The participants had to cruise in the third lane throughout the experiment. **(A)** The participants were required to cruise the third lane in the whole experiment. **(B)** Only the lane deviation was displayed in the single-task driving condition. **(C)** Only the mathematic equation was displayed in the single-task math condition. **(D)** Both the lane deviation and mathematic equation were simultaneously displayed. **(E)** The mathematic equation appeared 400 ms before the lane deviation. **(F)** The lane deviation appeared 400 ms before the mental equation.

In the lane-keeping task, the car linearly drifted either to the right (curb) or left (opposite lane) side while cruising in the third lane, as shown in Figure 1A (Huang et al., 2009). Each left or right drift can be considered as one deviation. The participants were instructed to direct the car back to the cruising lane (the third lane) quickly by turning the steering wheel after detecting the random deviation (Figure 1B). There was one sensor to monitor the angle of steering wheel and stored the changes of angles.

The other task in this experiment was the mental calculation task, which involved working memory and induced an increase in the workload (Hitch, 1978; Sammer et al., 2007). In this study, the calculation task was to validate an arithmetic equation as being correct or incorrect (Figure 1C). Here, the ratio of correct to incorrect equations was 1:1. Each equation was based on addition without carrying, and the difficulty level was manipulated to be similar across equations. Each equation was continuously displayed on the screen for 1200 ms. There were two buttons built on the steering wheel for responding to the numerical equation. The right (left) button was pressed with the thumbs if the equation was correct (incorrect).

The deviation and arithmetic equations were displayed simultaneously in the drive (D) and math (M) condition as shown in Figure 1D. To induce distinct attentional requirements, a 400-ms SOA was applied in this experiment. Jensen et al. (2001) have shown that humans require approximately 300 ms (P300 activity) to perceive a visual stimulus, and therefore, an SOA of 400 ms between two vision-based tasks is sufficient for participants in this distracted driving experiment. In the M/D condition, the numerical equation was displayed first, and the deviation appeared 400 ms after the onset of the mental calculation (Figure 1E). In the D/M condition, the deviation was displayed 400 ms before the onset of the arithmetic equation (Figure 1F).

A total of five conditions, two single tasks (D and M), one simultaneous dual task (D&M) and two dual tasks with a 400-ms SOA (M/D and D/M), were randomly displayed throughout the experiment. The inter-trial interval was between six to eight seconds. To ensure the participants’ awareness, they were instructed to undergo normal sleep, and the experiment was executed in the early morning (9:00 AM). Before the experiment, all participants were acquainted with the virtual-reality-based driving environment and the operation of the controls (turning the steering wheel and pressing the buttons) in two training sessions. After that, every participant was required to finish four 15-min sessions separated by 10-min breaks to prevent drowsiness during the experiment.

### Data Acquisition

The behavioral data, including the RT to the deviation and the solution time (ST) of the mental calculation, were recorded. There was a sensor in the steering wheel to collect the turning angle for the reaction to the deviation and a button to record the ST for providing an answer to the mental calculation. All information from the steering wheel was also synchronized with the designed scenario to provide real-time and suitable feedback to the participants.

EEG data were acquired by a 32-channel EEG cap (NuAmps, Compumedics NeuroScan, USA) at a sampling rate of 500 Hz with 16-bit precision. Thirty Ag/AgCl electrodes were arranged according to the modified international 10–20 system, and two reference electrodes (A1 and A2) were placed on both mastoid bones. The impedance between the electrodes and cortex was under 10 kΩ with a NaCl-based conductive gel.

### Data Analysis

#### Behavioral Data Analysis

The RT was defined as the latency between the onset of the deviation and the turning of the steering wheel. The latency from the presentation of the numerical equation to the button press was the ST. Both RTs and STs were behavioral indicators used to evaluate the performance. One-way ANOVA was independently performed to compare the mean STs for mental calculations or RTs for lane deviation among the single- and dual-task conditions. *Post hoc* Wilcoxon signed-rank tests were conducted for follow-up analysis. We also utilized one-way ANOVA and* post hoc* Wilcoxon signed-rank tests to analyze the accuracy of the lane-deviation correction and mental calculation during the single- and dual-task conditions.

#### EEG Pre-processing

All raw EEG data were pre-processed by applying finite impulse response (FIR) filters in a frequency range between 0.5 Hz and 50 Hz to remove the DC-drifts and high-frequency artifacts with re-sampling at 250 Hz. The continuous EEG data were segmented into a set of epochs according to the distinct conditions (Figure 1). For each single epoch, the continuous data length was from −1 s (prior to the first stimuli onset) to 5 s (following the first stimuli onset) to include all relevant activations. The epochs with large eye movements, body movements, and amplifier saturation were manually removed first.

#### Independent Component Analysis (ICA)

Independent component analysis (ICA) has been proposed for separating temporally independent activation and source identification (Jung et al., 1996). ICA, which is one method for EEG artifact removal with blind source separation, has been applied to remove a wide variety of artifacts from EEG records (Jung et al., 2001). Artifactual components accounting for eye blinks, eye movements, muscle activity and bad channels were isolated and removed (Jung et al., 1996). The independent components (ICs) were categorized into brain or non-brain (artifactual) activity based on the activity time course and projection to the recording electrode. Instead of channel data, the component data without artifacts were analyzed. In this study, 30 ICs were separated via ICA from the 30 channels of the EEG signals, which excluded the two reference electrodes (A1 and A2). All ICs with brain activity were grouped into several clusters by 2D scalp topographies of the mixing matrix, dipole source locations, and event-related spectral perturbations (ERSPs). The clusters that accounted for the frontal, parietal, and occipital components were selected.

#### Event-Related Spectral Perturbation (ERSP)

The purpose of ERSP is to observe the mean spectral changes as a function of the time associated with the cognitive stimuli. The activations in the selected ICs were transformed into power spectra through a fast Fourier transform (FFT) in a frequency range between 0.5–50 Hz. To investigate the perturbations associated with the stimuli or actions, the power spectra in the post-stimulus period (0–5 s) were normalized based on the averaged spectra in the pre-stimulus period (−1 to 0 s, baseline). The mean of each spectrum in the baseline was subtracted from each power spectral component after the onset of the first stimulus, and this procedure was applied to each single epoch. Then, the averaged spectral changes could be evaluated as a function of time, and a bootstrap analysis was also applied to each spectrum. The ERSP results were then depicted as a 2D time-frequency plane with the significant spectral differences compared to the baseline spectrum prior to the event onsets (0.05 for the bootstrap significance level).

The intention of analyzing the spectral perturbations sorted based on the behavioral data (RTs and STs) is to reveal the theta (4–7 Hz) and alpha (8–13 Hz) changes as a function of the specific events. The spectral perturbations in the frequency ranges of theta and alpha were extracted. In single-task conditions, all EEG epochs associated with deviation or mental calculation were sorted according to the RTs or STs, respectively. In dual-task conditions, the EEG epochs were sorted based on the STs. These sorted theta or alpha perturbations were then smoothed across epochs (Goh and Law, 2002).

#### Interactive Activations

Averaged theta and alpha perturbations in the selected components were extracted from the baseline-normalized and significant spectral dynamics. For each time point, the deduced spectral perturbations associated with the dual-task conditions were evaluated through comparison with the summation of the elicited spectral perturbations in the two single-task conditions. Since a 400-ms SOA was applied in the current study, the summation of the spectral perturbations was coordinated according to the dual-task conditions in this study. For example, in the D/M condition, the averaged theta and alpha dynamics extracted from single calculation were shifted 400 ms. Compared with the total activation of two single tasks, the higher, equal and lower brain oscillations associated with performing the dual-task conditions represent O-Add, Add and U-Add activation, respectively. For each time point, we used the Wilcoxon signed-rank test to test the pair-wise difference between the measured oscillations in the dual tasks and the summed oscillations associated with the two single tasks. To account for multiple comparisons, we then computed the false discovery rate (FDR)-adjusted *p*-values.

## Result

### Behavior

**Figure 2:**
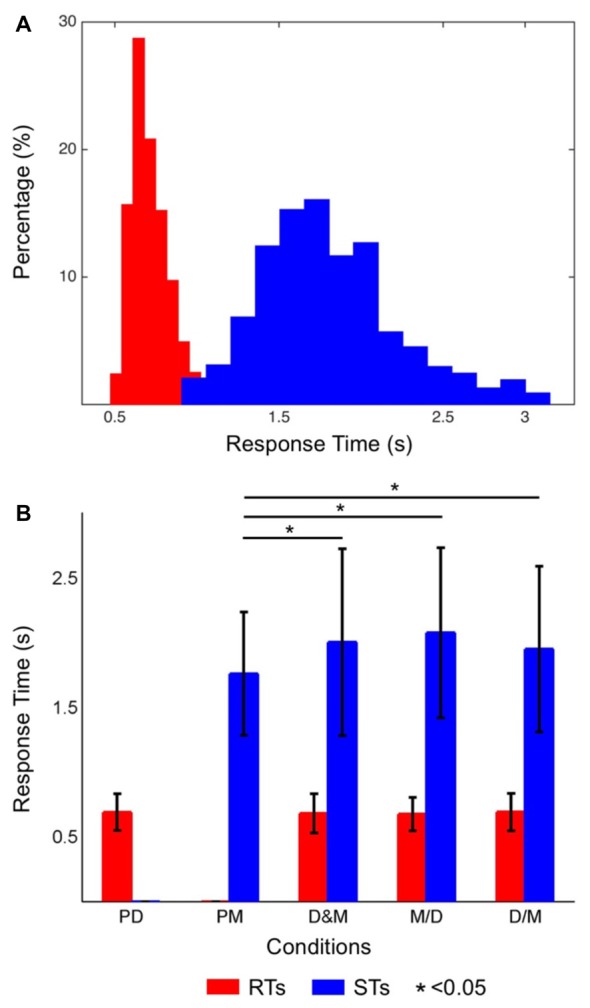
**(A)** Histograms of the reaction times (RTs; distributions of red bars) and solution times (STs; distributions of blue bars). The short RTs indicate that the participants had a high level of alertness throughout the experiment. **(B)** The averaged RTs of the driving task (red bars) and the STs of the math task (blue bars) in each condition. The STs were significantly different between the single- and dual-task conditions (*p* < 0.05), but the RTs were not significantly different between the single- and dual-task conditions.

Figure 2A shows the distribution of the RTs (red bars) and STs (blue bars) while only performing the lane-deviation and mental calculation tasks, respectively. The RTs of the driving task were in the range of 440–1188 ms. The participants required more time for the mental calculation, and the distribution of the STs was in the range of 834–2992 ms. Figure 2B shows the averaged RTs and STs, and the STs are significantly longer than the RTs (*p* < 0.05). The RTs in the dual-task conditions (D&M, M/D and D/M) were not significantly different from those in the single-task conditions. In contrast, the STs in all three dual-task conditions were longer than those in the single-task condition (*p* < 0.05). The accuracy for responding to both tasks in this experiment was 96.7 ± 1.4%. The differences in the error rates between the single- and dual-task conditions were not statistically significant.

### Brain Dynamics

#### Single Tasks

**Figure 3:**
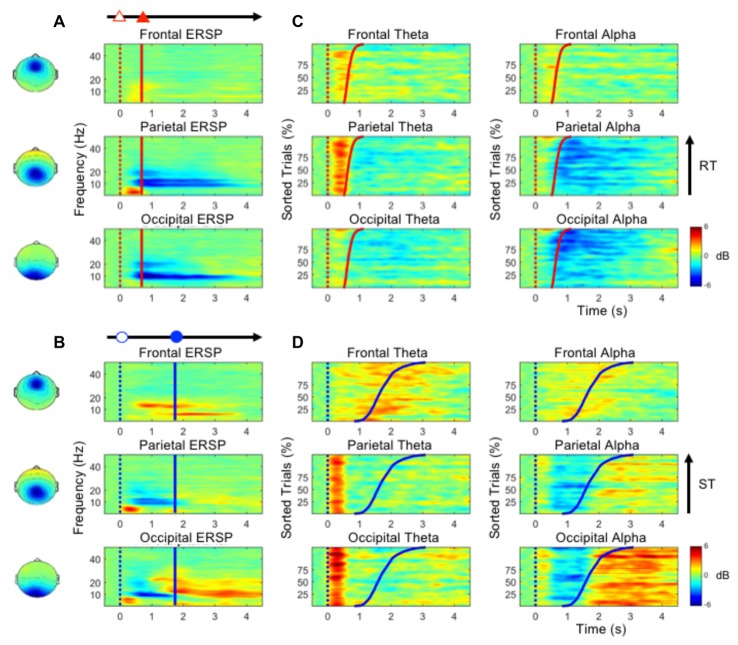
**(A,B)** The event-related spectral perturbation (ERSP) plots in the frontal, parietal and occipital components show the time-frequency results during the performance of the lane-deviation or mental calculation task. **(C,D)** The performance-related changes in the theta and alpha frequencies in the frontal, parietal and occipital components. The epochs with quick RTs or STs are depicted in the bottom of each sub-figure. The red and blue dotted lines indicate the onset of lane deviation and mental calculation, respectively. The red and blue solid lines indicate the onset of turning the steering wheel and the button-press response, respectively.

Figure 3A shows the ERSP plots in the frontal, parietal and occipital components during performance of the deviation task only (bootstrap, *p* < 0.05). While performing the lane-deviation task, both the frontal and parietal areas exhibited increased theta activity (Figure 3A). A decreased alpha power was found in the parietal and occipital components (Figure 3A). Figure 3C depicts the spectral perturbations in the theta and alpha frequency ranges according to the sorted RTs. The epochs with shorter RTs are at the bottom of the figure. As shown in Figure 3C, the theta bursts in the parietal components evidently followed the onset of the deviation (after the red dotted lines in Figure 3C). The decreased alpha oscillations in the parietal and occipital components began at the response onset (after the red lines in Figure 3C).

Figure 3B depicts the ERSP plots in the frontal, parietal, and occipital components during the execution of the mental calculation task (bootstrap, *p* < 0.05). Figure 3D depicts the spectral perturbations in the theta and alpha frequency ranges according to the sorted STs. An increased theta was pronounced across the frontal, parietal, and occipital components during the execution of the mental calculation task, and an increased alpha was observed in the frontal and occipital components (Figure 3B). However, a decreased alpha was observed in the parietal and occipital components during the calculations (Figure 3B). Frontal theta and alpha bursts were related to the calculations. In posterior areas, a sustained theta peaked after the onset of the numerical equation (after the blue dotted lines in Figure 3D) and was followed by a decreased alpha oscillation. Specifically, the brief theta and alpha oscillations were both time-locked to the onset of a mathematical equation (blue dotted lines in Figure 3D). The increased alpha oscillation following the button-press response was only observed in the occipital component (after the blue lines in Figure 3D). The stimulus- or response-related brain oscillations were summarized in Table 1.

**Table 1:** The brain oscillations while executing deviation or calculation.

Brain region	Oscillation	Task	Finding	Related with
Frontal	Theta	D	Slightly +	Onset of deviation
		M	+	Calculation
	Alpha	D		
		M	Slightly +	Calculation
Parietal	Theta	D	+	Onset of deviation
		M	+	Onset of numerical equation
	Alpha	D	−	Onset of reacting deviation
		M	−	Onset of numerical equation (following the increased posterior theta)
Occipital	Theta	D		
		M	+	Onset of numerical equation
	Alpha	D	−	Onset of reacting deviation
		M	−	Onset of numerical equation (following the increased posterior theta)
			+	Onset of finishing calculation

*+ increased activation, − decreased activation*.

#### Dual Tasks

**Figure 4:**
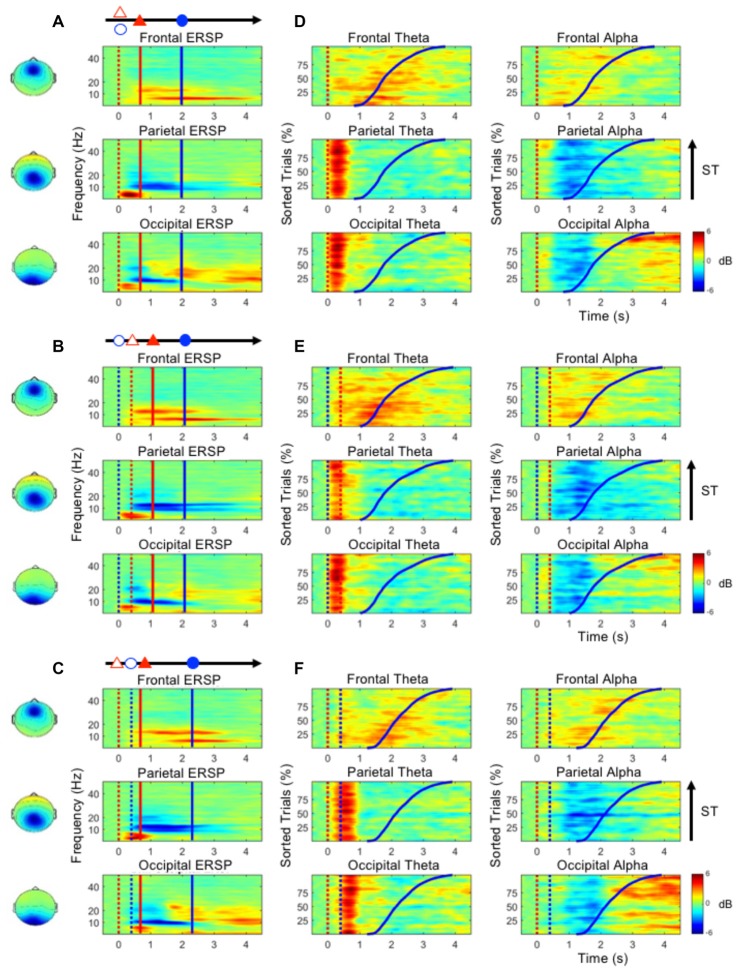
**(A–C)** The mean ERSP plots in the frontal, parietal and occipital components show the time-frequency results during the performance of the dual-task conditions. **(D–F)** The performance-related changes in the theta and alpha frequencies in the frontal, parietal and occipital areas. The epochs with quick STs are depicted in the bottom of each sub-figure. The red and blue dotted lines indicate the onset of lane deviation and mental calculation, respectively. The red and blue solid lines indicate the onset of turning the steering wheel and the button-press response, respectively.

Figures 4A–C show the ERSP plots as executing the three dual-task conditions, the D&M, M/D and D/M conditions (bootstrap, *p* < 0.05). Figures 4D–F depict the dynamic changes in the theta and alpha bands according to the sorted STs. An increased frontal theta oscillation is mainly observed around the onset of the button-press (the blue lines in Figure 4). Figure 4 shows that an increase in the parietal theta oscillation is pronounced following the first stimulus (either lane deviation or numerical equation) in the three dual-task conditions. In the occipital component, an increased theta oscillation is only observed following the onset of the numerical equation (blue dotted lines in Figures 4B–F) and not with the onset of the lane deviation (red dotted lines in Figures 4D,E). This occipital theta burst is in a range of 0–500 ms in the D&M and M/D conditions. In the D/M condition, the increased occipital theta oscillation associates with the onset of the numerical equation occurred from 400 ms to 1 s.

During the execution of the dual-task conditions, an increased theta oscillation in the frontal component and decreased alpha oscillations in the parietal and occipital components are observed (Figure 4). In the parietal and occipital components, the decreased alpha is pronounced following the increased theta activation (Figure 4). In particular, the increased occipital alpha oscillation is majorly observed in the epochs with long STs (after the blue lines in Figure 4).

### The Interactive Activation between Single and Dual Tasks

**Figure 5:**
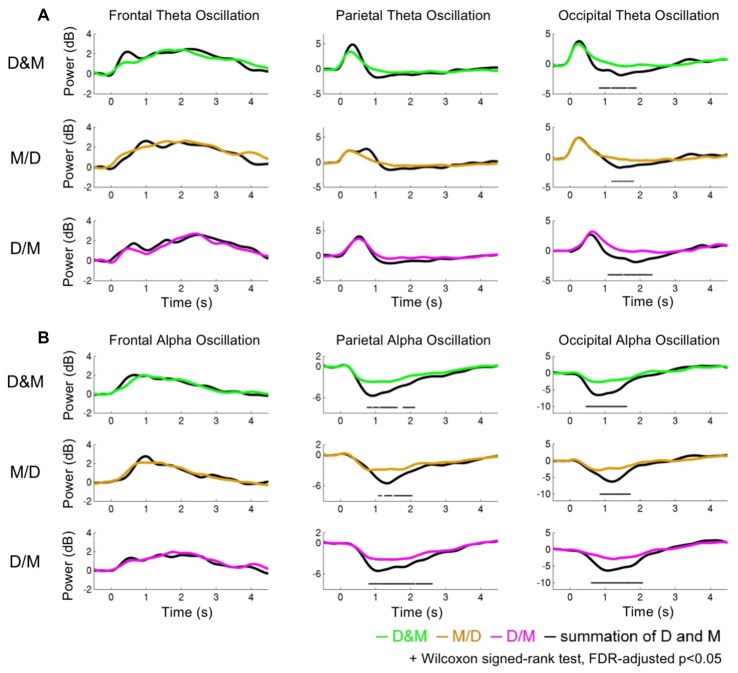
The dynamics of the theta and alpha oscillations. **(A)** The theta activations **(B)** and alpha activations. The green, yellowish brown and pink lines indicate the recorded oscillations during the dual conditions: simultaneous (D&M), calculation first (M/D) and deviation first (D/M), respectively. The black lines represent the interactive activations, which are the sum of the activations while executing the two tasks individually.

Figure 5 shows the theta and alpha oscillations as a function of time in the frontal, parietal and occipital components while performing the D&M (green lines), M/D (yellowish brown lines) and D/M (pink lines) conditions. The black lines represent the sum of the brain activations induced by executing deviation and calculating. The O-Add, Add and U-Add activations induced by performing the dual-task conditions represent the activations higher than, equal to and lower than the summed activation, respectively. The comparison between the recorded activation and the simulated activation are marked (Wilcoxon signed-rank test, FDR-adjusted *p* < 0.05).

Figure 5A depicts the comparison of the theta activation. In the frontal area, the O-Add activation is only observed in the D&M condition, and Add activation is found in the M/D and D/M conditions. The Add activation of the parietal theta oscillation is observed in the M/D and D/M conditions, but U-Add parietal theta activation is only found in the D&M conditions. In the occipital region, the Add theta activation is first found, followed by the significant U-Add theta activation.

Figure 5B shows the comparison of the alpha activations in the frontal, parietal and occipital components. The Add frontal alpha activation is observed in all three dual-task conditions. In the parietal and occipital components, the significant U-Add alpha activation dominates during the D&M, M/D and D/M conditions. In addition to the findings in Figure 5, Table 2 summarizes the interactive theta and alpha activations in these three dual-task conditions.

**Table 2:** The brain dynamics of interactive attention in the three dual-task conditions.

Brain region	Oscillation	Condition	Activation
Frontal	Theta	M&D	O-Add
		M/D	Add
		D/M	Add
	Alpha	M&D	Add
		M/D	Add
		D/M	Add
Parietal	Theta	M&D	U-Add
		M/D	Add
		D/M	Add
	Alpha	M&D	U-Add*
		M/D	U-Add*
		D/M	U-Add*
Occipital	Theta	M&D	Add, U-Add*
		M/D	Add, U-Add*
		D/M	Add, U-Add*
	Alpha	M&D	U-Add*
		M/D	U-Add*
		D/M	U-Add*

*Wilcoxon signed-rank test, FDR-adjusted p < 0.05.

## Discussion

The RTs in the lane-deviation task throughout the experiment imply that the participants maintained their awareness (Figure 2A). The STs were significantly longer in the three dual-task conditions than in the single-task condition (Figure 2B). Yerkes and Dodson (1908) demonstrated that multitasking and attention switching indeed lead to decreased behavioral performance, even when subjects are in a high state of arousal (Diamond et al., 2007). Decreased mental calculation performance reflects an increased workload, divided attention, and an overlap in resources used during dual-task situations (Salvucci and Taatgen, 2008).

Due to the increased RTs observed in previous dual-task studies, greater brain activation should be expected during the performance of two tasks simultaneously (Szameitat et al., 2011). This study demonstrated interactive theta and alpha activations during the dual tasks. Table 2 lists that an Add theta activation is primarily observed in the selected components during the dual-task conditions except D&M. The O-Add frontal theta and U-Add parietal theta activations are in the D&M condition. Meanwhile, the Add frontal alpha and U-Add posterior alpha activations are observed during dual-task situations. In particular, there are significant difference in the U-Add occipital theta and posterior alpha in these three dual conditions (Wilcoxon signed-rank test, FDR-adjusted *p* < 0.05).

### Theta Oscillations

In Figure 5A and Table 2, a non-significant O-Add frontal theta activation was only observed in the D&M condition. In Jaeggi’s study, slight O-Add activation during the simultaneous performance of two working memory tasks (1-back and 2-back) was primarily found in the frontal cortex, which is involved in cognitive processes and the management of concurrent processes (Jaeggi et al., 2003; Dux et al., 2006). The O-Add frontal theta activation in the D&M condition suggests that extra activation was required to perform the tasks. Enhanced frontal theta oscillations have been positively correlated with increased task demands and workload (Klimesch, 1999; Lin et al., 2011; Borghini et al., 2014) as well as decision-making, orienting, conditioning, numerical calculation, workload, working memory and the maintenance of goal states (Gevins et al., 1997; Klimesch, 1999; Sauseng et al., 2005; Kahana, 2006; Sammer et al., 2007; Tombini et al., 2009; Huang et al., 2013). Compared with performing the lane-deviation or mental calculation tasks alone, the extra demands due to attention switching and decision making must be expected. However, Add frontal theta activation was observed in the M/D and D/M conditions (Figure 5 and Table 2). Since a 400-ms SOA was applied in the M/D and D/M conditions, the participants might have been able to optimize their performance in the lane-deviation and mental calculation tasks without extra attentional requirements. The different findings in the frontal theta represent the distinct attentional requirements in three designed dual conditions. These non-significant O-Add or Add frontal theta activations suggest that the extract attention are slightly required as managing lane deviation and mental calculation simultaneously. It means the degree of difficulty of the designed tasks are medium.

Furthermore, U-Add parietal theta activation was only found in the D&M condition after the event onset, but Add parietal theta activation was found in the M/D and D/M conditions (Figure 5 and Table 2). Enhanced parietal theta activation has been shown to modulate the strength of the sensory (Raghavachari et al., 2006). In the current study, two visual stimuli were displayed simultaneously to the participants, and the tasks were displayed sequentially in the M/D and D/M conditions with a 400-ms SOA. The U-Add and Add parietal theta activation suggests that the two visual stimuli can be processed with time-sharing resource allocation (Nijboer et al., 2014). Therefore, U-Add parietal theta activation was expected in the D&M condition.

In the occipital region, Add activation and U-Add activation were both observed in the dual-task conditions. In addition to the time-sharing requirement discussed above, Add activation could also be explained by a specific brain oscillation only induced by one of the tasks (Salvucci and Taatgen, 2008). Increased occipital theta activation was only observed in the early stages of mental calculation (Figures 3B,D), and occipital theta activation has been responsible for controlling cognitive demands (Raghavachari et al., 2006). Specifically, identifying and processing stimuli is associated with occipital activation (Cartier et al., 2012). The increased occipital theta oscillation at the beginning of the mental calculation can be interpreted as a high cognitive demand in response to visual stimuli. Since the increased occipital theta activation was elicited by mental calculation alone (Figure 3B, Table 1 and Figure 4), Add activation was expected. In particular, this occipital theta oscillation was clearly observed after the onset of the mental calculation in the M/D and D/M conditions (the red dotted lines in Figures 4B,C). Following the Add occipital theta activation, a significant U-Add theta activation was then observed in the occipital component. The occipital theta activation was only slightly higher than that observed during the performance of the lane-deviation task (Figure 3). During the dual-task conditions, the U-Add theta activation represents lower resource requirements. This U-Add occipital theta activation is similar to the U-Add alpha activation in the posterior region.

### Alpha Oscillations

In Table 2, non-significant Add alpha activation was observed in the frontal component, and significant U-Add alpha activations were found in the posterior region (Wilcoxon signed-rank test, FDR-adjusted *p* < 0.05). Like the theta activation in the frontal area, the posterior alpha is also associated with cognitive processes and attentional demands (Klimesch et al., 1998; Foxe and Snyder, 2011; Lai et al., 2012; Lin et al., 2012a). Due to resource competition between tasks, U-Add activation occurs when one task dominates most of the resources used (Anderson et al., 2011). Decreased posterior alpha activation is associated with visual scanning (Fu et al., 2001). In the current study, reacting to the lane deviation suppressed posterior alpha activation (Figure 3A). Participants were required to continuously focus on the vehicle position with respect to the center of the cruising lane during the lane-keeping task. The posterior alpha suppression could also be attributed to an increased workload and short-term memory requirements during mental calculation (Gevins et al., 1997; Meeuwissen et al., 2011). While performing the mental calculation, participants visually scanned the equation and retrieved numerical meanings to obtain the correct answer for the current equation. Compared with the lane-keeping task, mental calculation is a task that requires more cognitive functions and induces a higher workload. In particular, the averaged STs were significantly longer than the averaged RTs. During the simultaneous performance of the lane-deviation and mental calculation tasks in the dual-task conditions, paying more attention to the mental calculation led to the significant O-Add activation in the posterior area.

In the frontal region, an Add alpha activation was primarily observed in the dual-task conditions (Figure 5 and Table 2). As discussed above, the brain oscillation related to only one task leads to Add activation. Increased frontal alpha activation has been correlated with internally directed attention (Travis and Shear, 2010). Previous working memory studies have reported that increased frontal alpha activation involves in accurately retrieving memorized information (Klimesch et al., 2005; Huang et al., 2013). In the current study, the increased alpha in the frontal cortex was induced while performing numerical calculations (Figures 3B,D). This increased frontal alpha oscillation before the button-press response could be attributed to the fact that participants had to memorize the displayed numerical equation and compare it with the answer while making a mental calculation. The Add frontal alpha activation was expected since the increased frontal alpha activation was only involved in one task (mental calculation).

## Conclusion

In the current study, two visual stimuli, lane deviation and mental calculation, were utilized to explore the behavioral changes and brain dynamics in the frontal, parietal and occipital regions during the performance of dual tasks. The impaired STs of the mental calculation reflected the attentional interaction and overlapping resources used during the dual-task situations. The time-varying interactive O-Add, Add and U-Add activations were explored using dual tasks with distinct attentional requirements. Rather than O-Add activation in the human brain, Add and U-Add activations were primarily identified in this dual-task study with a 400-ms SOA. During the performance of dual tasks, O-Add activation was expected due to the impaired behavior, indicating the need for more attention for switching tasks and balancing performance results in O-Add activation. However, this higher oscillation was only observed in the frontal theta dynamics during the D&M condition. Perfect time-sharing integration and only one task-related activation lead to Add activation. This type of activation was primarily observed in frontal theta, frontal alpha, parietal theta and occipital theta activation. Competition between different tasks and limited resources result in U-Add activation, which was observed in posterior alpha and occipital theta oscillations.

Past brain-computer interface studies have tried to model the increased workload and impaired attention from the activation in the frontal or posterior region. According to the results from three different dual-task conditions with a 400-ms SOA, these empirical findings support the observation that brain dynamics during multitasking are a complex composition of the activation induced by each single task. The study opens a new window into the use of EEG spectra analysis to evaluate attentional interaction during the execution of dual or multiple tasks.

## Author Contributions

T-PJ and C-TL: conceived and designed the experiments. Y-KW and C-TL: performed the experiments. Y-KW and T-PJ: analyzed the data. Y-KW, T-PJ and C-TL: wrote the article.

## Conflict of Interest Statement

The authors declare that the research was conducted in the absence of any commercial or financial relationships that could be construed as a potential conflict of interest.
